# Trauma-Informed Care on CAMHS ID Unit. Case Study of a Child with Neurodevelopmental Disorder and Self-Injurious Behaviour

**DOI:** 10.1192/bjo.2022.356

**Published:** 2022-06-20

**Authors:** Evelina Eggleston, Omaima Daoud

**Affiliations:** College of North West London, The Kingswood Centre, London, United Kingdom

## Abstract

**Aims:**

Crystal House is a specialist CAMHS ID inpatient 5-bedded unit based in the Kingswood Centre, North West London - for children aged between 13 and 18 years with primary diagnosis of Intellectual Disabilities with or without additional concerns that warrant admission to hospital for purpose of assessment and management. Reporting this case, we wanted to highlight complexities of management of children presenting with neurodevelopmental conditions and history of trauma.

**Methods:**

This is a case of a fourteen-year-old girl with established diagnoses of Moderate Intellectual Disability, Childhood Autism, Foetal Alcohol Syndrome and childhood trauma. She was admitted to our CAMHS ID Assessment and Treatment Unit with a nine- year history of self-injurious behaviour, suicidal ideation and voice-hearing experiences – after failed treatment in the community and in-patient treatment (including under restrictions of long-term segregation) on generic CAMHS unit. Her current treatment includes a person-centred Trauma- Informed Positive Behaviour Support Plan; individual and family therapy psychology sessions based on the principles of trauma-informed care and consultation with staff on trauma-informed care. She also undertook ADHD assessment, and we are in the process of optimising ADHD medication.

**Results:**

Trauma-Informed Positive Behaviour Support Plan was a new concept for the team. Therefore, this was supported by training and consultations with staff. The latter was introduced to create a psychologically oriented environment using trauma-informed care principals and helping the team understand what trauma means and how it affects the individual. ADHD assessment confirmed the diagnosis of ADHD which was followed by optimising ADHD medication.

**Conclusion:**

Systemic and a person-centred approach is used for this child with concerning presentation and history of neurodevelopmental disorder and childhood trauma.

